# Applied Anatomy of Bulbospongiosus Muscle: a Narrative Review

**DOI:** 10.1590/S1677-5538.IBJU.2025.9917

**Published:** 2025-09-30

**Authors:** William Lannes, Jorge L. Alves-Pereira, Eduardo M. Ribeiro, Edilaine F. Alves, Luciano A. Favorito

**Affiliations:** 1 Universidade do Estado do Rio de Janeiro Unidade de Pesquisa Urogenital Rio de Janeiro RJ Brasil Unidade de Pesquisa Urogenital - Universidade do Estado do Rio de Janeiro - Uerj, Rio de Janeiro, RJ, Brasil; 2 Universidade Estácio de Sá Instituto de Educação Médica Rio de Janeiro RJ Brasil IDOMED – Instituto de Educação Médica, Universidade Estácio de Sá - UNESA, Rio de Janeiro, RJ, Brasil

**Keywords:** Anatomy, Muscles, Ejaculatory Dysfunction

## Abstract

This review aims to provide a comprehensive analysis of the anatomy of the bulbospongiosus muscle (BSM) in males, highlighting its morphological features, anatomical variations, and clinical implications in ejaculatory process and urethral surgery. We analyzed papers published in the past 70 years in the databases of Pubmed, Embase and Scielo. We excluded case reports, editorials and opinions of specialists. Studies were excluded if they lacked clear anatomical descriptions of the BSM, if full-text access was unavailable, or if they were published in languages other than Portuguese, English, or Spanish. The BSM is part of the superficial muscular layer of the perineum The perineal arteries provide vascular supply to BSM and, toward the end of their path, give rise to several posterior scrotal branches that supply the skin and dartos muscle of the scrotum. The deep branch of pudendal nerve is primarily muscular and innervates the superficial transverse perineal, bulbospongiosus, ischiocavernosus, deep transverse perineal, and sphincter muscles of the membranous portion of the urethra. This revision concluded that the BSM is a paired skeletal striated muscle that originates from the perineal body enveloping the bulbar portion of the urethra. Stimulation of the dorsal nerve of the penis leads to BSM contraction that provides an indication of anatomic sacral nerve pathway integrity. BSM has some important clinical applications. The bulbar urethral stricture causes minimal alterations in the structure of the BSM during the surgical procedure and the BSM is highly sensitive to androgen and could be used in common androgenic activity evaluation.

## INTRODUCTION

The perineum represents the region forming the inferior boundary of the pelvic cavity, with well-defined deep anatomical landmarks: anteriorly, it is limited by the pubic arch and the arcuate pubic ligament; posteriorly, by the most inferior and prominent portion of the coccyx; and laterally, by the inferior rami of the pubis and ischium, along with the sacrotuberous ligament. On the surface of the body, the perineal region presents a diamond-shaped contour, bounded anteriorly by the scrotum (in males), posteriorly by the gluteal region, and laterally by the medial aspects of the thighs (1-3).

The superficial perineal space, located more inferiorly, is separated from the subcutaneous adipose tissue by the superficial perineal fascia. Posteriorly, this fascia merges with the adipose tissue of the ischioanal fossa, while anteriorly, it continues as the superficial fascia of the abdomen. Structures within this space include the superficial transverse perineal muscle, the bulbospongiosus muscle (BSM), the ischiocavernosus muscle, the bulb of the penis (in males), the vestibule of the vagina (in females), the crura of the corpora cavernosa of the penis (in males), but are not prolonged into the penile shaft, clitoris and its glans (in females), as well as the terminal branches of the internal pudendal vessels and pudendal nerve (1-3).

Bulbospongiosus muscle (BSM) is a paired striated muscle originating from the perineal body and fixed in a median raphe that involves the bulbar urethra and its function is the expulsion of seminal fluid and urine from the urethra. The BSM is a component of the superficial perineal musculature and is located within the superficial perineal space, surrounding the bulb of the penis in males and the vestibular bulbs in females. Anteriorly, it is closely associated with the ischiocavernosus muscle, posteriorly its dorsal fibers may establish variable connections with the external anal sphincter. BSM acts as a pump to expel seminal fluid and urine (last drop) in bulbar urethra by rhythmic contractions (4-6).

Given the anatomical and functional relevance of the structures within the perineal region, particularly the superficial perineal space, this review aims to provide a comprehensive analysis of the anatomy of the BSM in males, highlighting its morphological features, anatomical variations, and clinical implications in ejaculatory process and urethral surgery.

## MATERIAL AND METHODS

In this study we carried out a narrative review about the anatomy of the bulbospongiosus muscle (BSM) and its anatomical and clinical relevance in the male population. We analyzed papers published in the past 70 years in the databases of Pubmed, Embase and Scielo, found by using the key expressions: "bulbospongiosus muscle", "bulbocavernosum muscle", "male pelvis", "perineum", "urethroplasty", "ejaculatory dysfunction" and "urological relationships".

Inclusion criteria comprised original articles, anatomical and clinical studies, imaging-based investigations, and both narrative and systematic reviews addressing the morphological, functional, or clinical aspects of the BSM in men. In this review we excluded case reports, editorials and opinions of specialists. Studies were excluded if they lacked clear anatomical descriptions of the bulbospongiosus muscle, if full-text access was unavailable, or if they were published in languages other than Portuguese, English, or Spanish.

## RESULTS

### Embriology of Bulbosponjosum Muscle

**Figure 1:**
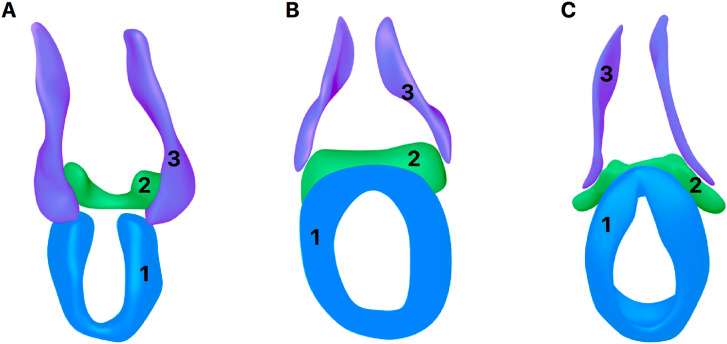
Embriology of Bulbospojosum muscle (BSM).

The BSM originates from the mesenchyme of the perineal region, forming part of the superficial musculature of the perineum during fetal development (Figure-1). Mapping studies have provided remarkable evidence that the ventral muscle mass of the hind limb also contributes to the formation of the perineal musculature. This group includes the external anal sphincter, the superficial transverse perineal muscle, the ischiocavernosus muscle, the BSM, the deep transverse perineal muscle, and the external urethral sphincter. During embryogenesis, precursor cells of these muscles migrate caudally from the hind limb buds toward the developing perineal region, where they differentiate and become functionally organized (7, 8).

Schematic drawings based in the paper of Hulsmman (8).

A) The cutaneous external anal sphincter (1) and bulbospongiosus muscle (2) can be identified at 8 weeks post conception; B) The superficial transverse perineal muscle (3) is identifiable at 9 weeks post conception and C) at the 10 weeks post-conception we can observe the ventral closure of the external anal sphincter. The BSM at this stage exhibit ventral projections, while the superficial transverse perineal muscles occupy a predominantly transverse orientation.

At embryonic week 8 (Carnegie Stage 23), a pre-muscular mesenchymal condensation can be observed in the region corresponding to the developing bulbospongiosus and external anal sphincter muscles. At this stage, these structures are not yet clearly distinguishable, forming a ventrally open, U-shaped mesenchymal condensation surrounding both the urogenital and anal outlets. The superficial transverse perineal muscle also becomes identifiable at this time, appearing as a radially oriented structure located between the primordial bulbospongiosus and external anal sphincter muscles. By week 9, a distinct narrowing emerges between the condensations of the bulbospongiosus and external anal sphincter, enabling clearer anatomical delineation (7, 8). From week 10 onward, ventral closure of the external anal sphincter occurs, establishing a true sphincter morphology. The BSM at this stage exhibit ventral projections, while the superficial transverse perineal muscles occupy a predominantly transverse orientation between them (8).

Although all three muscles can be anatomically distinguished from one another, they remain poorly defined in the midline of the pelvic floor, even by week 20 of development. The superficial transverse perineal muscle extends laterally but does not insert into the ischial bone; instead, it terminates within the surrounding pelvic mesenchyme. Cross-sectional analysis of the external anal sphincter reveals an elongated profile whose long axis gradually rotates from a perpendicular (∼90°) orientation relative to the transverse plane at week 7 to a predominantly horizontal (0°) alignment by week 20 (8). In Figure-1 we can observe the anatomical position of BSM in a male fetuses with 30 weeks post conception.

### Macroscopic Anatomy of Bulbosponjosum Muscle

**Figure 2:**
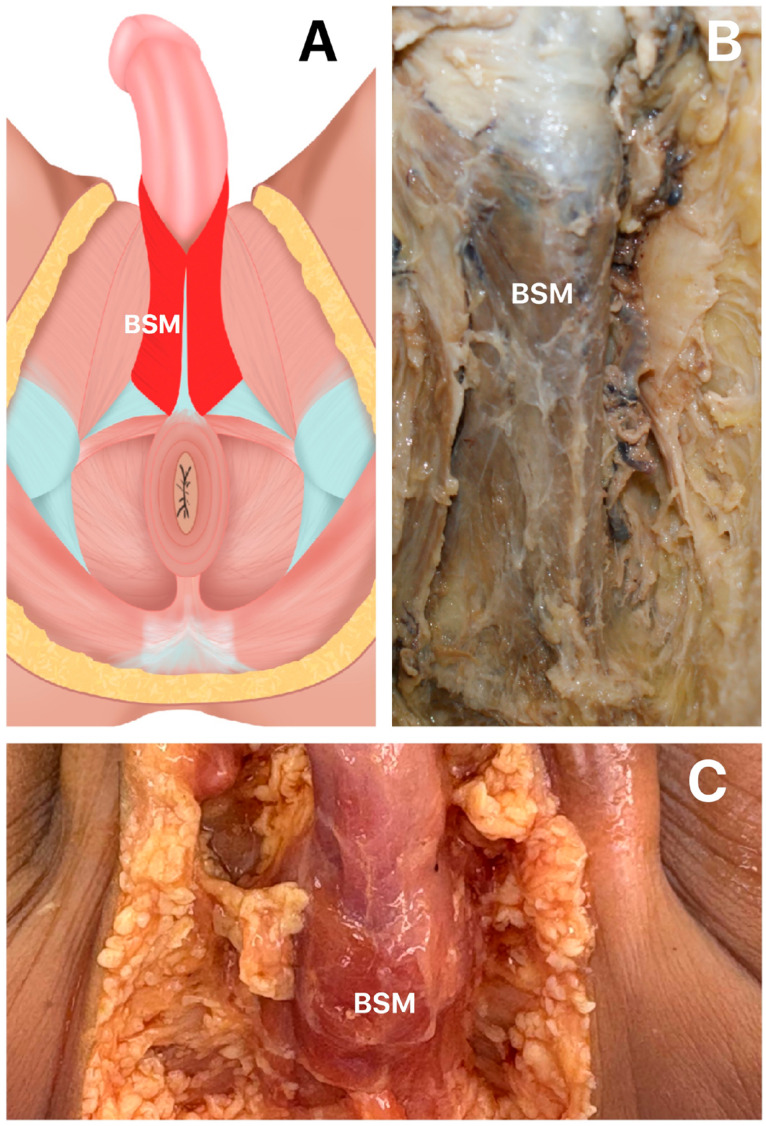
Macroscopic anatomy of Bulbospojosum muscle (BSM).

The BSM is a paired skeletal striated muscle that originates from the perineal body and inserts along a median raphe, externally enveloping the bulbar portion of the urethra (9). Also referred to as "musculus bulbocavernosus", is located along the midline of the perineum, ventral to the anus. It consists of two symmetrical parts, joined along the median plane by a tendinous raphe. It originates from the centrum tendineum perinei (central tendon of the perineum) and its anterior extension along the median raphe (Figure-2).

A) Schematic drawing showing the position of BSM in male perineum. The BSM can be observed in red and we can see the median raphe of the muscles. B) We can observe the BSM in a male adult fixed cadaver and C) we can observe the BSM in a fresh human male fetuses with 30 weeks post-conception.

Its fibers diverge like the barbs of a feather: the most posterior fibers form a thin layer that merges into the superficial fascia of the diaphragma urogenitale; the intermediate fibers encircle the corpus spongiosum penis and adjacent structures, and interlace with fibers from the contralateral side over the dorsum of the corpora cavernosa penis, forming a robust aponeurosis; the anterior fibers dissociate and spread over the lateral surfaces of the corpora cavernosa, inserting partially into them, anterior to the musculus ischiocavernosus. Occasionally, some fibers extend toward the os pubis and terminate in a tendinous expansion that invests the dorsal vessels of the penis. These terminal fibers can be visualized by dividing the muscle longitudinally and reflecting it from the surface of the corpus spongiosum penis.

The BSM is part of the superficial muscular layer of the perineum (10), and its morphology remains a subject of debate in scientific literature. Due to its function during erection and the activity of the BSM after urination and ejaculation to expel the last drops of urine and semen, the perineal muscles are generally more developed in men than in women.

In men, it originates from the median raphe on the ventral surface of the bulb of the penis in the perineal body. It follows a path that encircles the lateral surfaces of the bulb of the penis and the most proximal part of the penis body, inserting into the perineal membrane, the dorsal surface of the corpora spongiosum and cavernosa, and the fascia of the bulb of the penis.

Functionally, the BSM assists in emptying the urethral canal following bladder voiding. During most of micturition, the muscle remains relaxed, contracting only toward the end of the process. The intermediate fibers are believed to aid erection of both the corpus cavernosum and urethra by compressing the erectile tissue of the bulb. The anterior fibers also contribute to penile erection by compressing the penile veins as they insert into the fascia penis (6, 9, 10).

### Bulbosponjosum Muscle Vascularization

The perineum is irrigated by the two internal pudendal arteries, branches of the internal iliac (hypogastric) artery. The internal pudendal artery is the primary source of blood supply to the perineum and external genital organs. Although its general course is similar in both sexes, the vessel is typically smaller in females, and the distribution of its branches may exhibit slight anatomical differences between males and females (11, 12).

In males, the artery descends caudally toward the inferior border of the greater sciatic foramen, exiting the pelvis between the piriformis and coccygeus muscles. It then crosses the ischial spine and re-enters the pelvis through the lesser sciatic foramen. From there, it courses along the surface of the obturator internus muscle, which forms the lateral wall of the ischioanal fossa, gradually approaching the margin of the inferior ischiopubic ramus. The artery then travels anteriorly between the two layers of the urogenital diaphragm fascia and continues along the medial border of the inferior pubic ramus (11-13). Approximately 1.25 cm posterior to the arcuate ligament of the pubis, it bifurcates into the dorsal and deep arteries of the penis. In some cases, this bifurcation may occur before the artery pierces the superficial fascia of the urogenital diaphragm.

Topographically, within the pelvis, the internal pudendal artery is located anterior to the piriformis muscle, the sacral nerve plexus, and the inferior gluteal artery. As it crosses the ischial spine, it lies deep to the gluteus maximus muscle and is covered by the sacrotuberous ligament. At this point, the pudendal nerve is located medially, and the nerve to the obturator internus lies laterally. In the perineum, the artery runs along the lateral wall of the ischioanal fossa, within Alcock's canal, formed by the splitting of the obturator fascia. It is accompanied by paired veins and the pudendal nerve (11-13).

Several anatomical variations have been described. In some individuals, the internal pudendal artery is hypoplastic or fails to give rise to one or more of its typical branches. In such cases, the deficit is compensated by an accessory pudendal artery, usually originating from the internal pudendal artery before it exits the greater sciatic foramen. This vessel courses anteriorly along the inferior surface of the urinary bladder and the lateral aspect of the prostate to reach the root of the penis, where it pierces the urogenital diaphragm and gives rise to branches that would normally stem from the internal pudendal artery. The most frequently observed variation involves the internal pudendal artery terminating as the artery to the bulb of the urethra, with the dorsal and deep arteries of the penis arising from the accessory pudendal artery (11-13). In other cases, the internal pudendal artery may terminate as the perineal artery, while the artery to the bulb and other perineal branches originates from the accessory vessel, which may itself arise from branches of the internal iliac artery, most commonly the inferior vesical or obturator arteries.

Among the branches of the internal pudendal artery are: muscular, inferior rectal, perineal, artery of the bulb of the urethra, urethral, deep artery of the penis, and dorsal artery of the penis. Those directly involved with the bulbospongiosus muscle are of anatomical relevance in this context.

**Figure 3:**
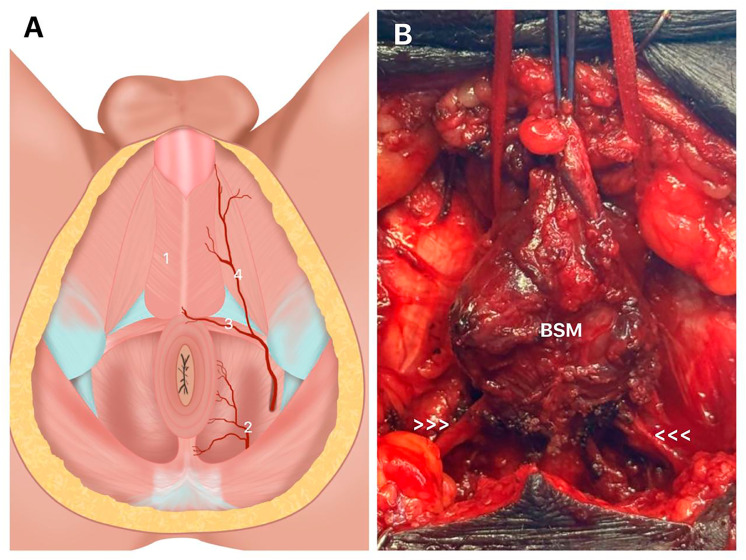
Arteries of Bulbospojosum muscles (BSM).

The perineal artery, one of the first branches of the internal pudendal artery upon entering the perineum, courses anterior to the superficial transverse perineal muscle, which it may cross either superiorly or inferiorly (Figure-3). It follows the pubic arch, running through the anatomical space between the bulbospongiosus and ischiocavernosus muscles. This artery provides vascular supply to both muscles and, toward the end of its path, gives rise to several posterior scrotal branches that supply the skin and dartos muscle of the scrotum.

A) We can observe a schematic drawing showing the distribution of perineal arteries in male. The internal pudendal artery has important branches and in this figure we can observe the inferior rectal artery (2), the perineal artery (3) and the continuation of the pudendal artery (4) that origins the artery of the bulb of the urethra, directly involved with the bulbospongiosus muscle irrigation. B) We can observe during a urethroplasty the bulbospojosum muscle (BSM) and the bulbourethra artery that is originated from the perineal artery (3).

Histological studies have shown that the vascular density of the bulbospongiosus muscle can be quantitatively assessed, and that pathological conditions such as bulbar urethral stricture are associated with a significant reduction in the number of intramuscular vessels, even in the absence of changes in other structural elements such as muscle fibers or collagen content. This finding emphasizes the critical role of vascular integrity in the functional maintenance of the muscle (9, 11, 12).

Anatomical literature further describes that vessels supplying the bulbospongiosus often travel alongside perineal nerve branches, highlighting the neurovascular complexity of the region. The penile vascular architecture is organized into multiple arterial axes and local neurovascular anastomoses. Therefore, the vascularization of the bulbospongiosus muscle is predominantly ensured by bulbar and urethral branches of the internal pudendal artery, with additional support from perineal vascular plexuses and anastomotic networks that help maintain an adequate blood supply, even under pathological conditions (9, 11, 12, 14).

### Bulbosponjosum Muscle Innervation

The most important nerve of perineum is the pudendal nerve. The pudendal nerve originates from the second, third, and fourth sacral nerves (S2-S4), and passing between the piriformis and coccygeus muscles, it exits the pelvis through the distal part of the greater sciatic foramen (15, 16). It then crosses the ischial spine and re-enters the pelvis through the lesser sciatic foramen. It accompanies the internal pudendal vessels along the lateral wall of the ischiorectal fossa, in the tunnel formed by a division of the obturator fascia (Alcock's canal). As it approaches the urogenital diphragm, its terminal branches divide into (15-17):

a) inferior rectal nerves: supply the external anal sphincter muscle and the perianal skin; b) perineal nerves: posterior scrotal/labial nerves, supply the skin of the external genitals; muscular branches, for the supply of the perineal muscles (deep and superficial transverse perineal muscles, bulbospongiosus muscle, ischiocavernosus muscle) and c) dorsal nerve of the penis/clitoris, for the sensory supply of the penis or clitoris.

The perineal nerve, the largest and most superficial of the terminal branches of the pudendal nerve, accompanies the perineal artery and, at the level of the urogenital diaphragm, divides into superficial and deep branches (14-17).

The deep branch of pudendal nerve is primarily muscular and innervates the superficial transverse perineal, bulbospongiosus, ischiocavernosus, deep transverse perineal, and sphincter muscles of the membranous portion of the urethra.

The penis is innervated by the dorsal penile nerves, which are branches of the pudendum nerve, in turn innervating the skin and mainly the glans. The deep branches of the perineal nerves, which enter the bulb and mainly innervate the urethra and cavernous penile nerves, in turn are branches of the inferior hypogastric plexus, responsible for the autonomic innervation of the penis, particularly the penile erectile bodies (18).

The inferior hypogastric plexus, containing sympathetic and parasympathetic nervous system fibers, is largely responsible for innervating structures associated with urinary continence as well as sexual function (15-17). In the periprostatic region, these fibers are in close relation to various vascular structures, and the whole complex is often termed the neurovascular bundle.

**Figure 4:**
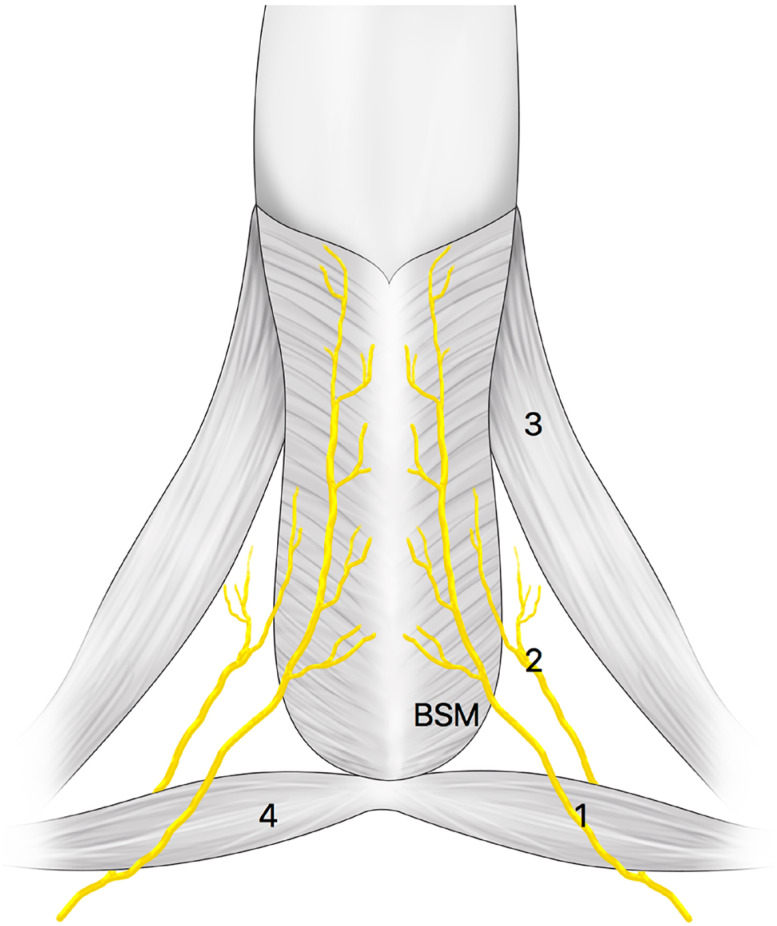
Nerves of Bulbospojosum muscles (BSM).

The perineal nerves are responsible to the BSM innervation. The perineal nerves, which are branches of the pudendal nerve, course along the bulbospongiosus and emit fine branches that penetrate the muscle and extend into the corpus spongiosum, particularly at the junction between the muscle and the erectile tissue (Figure-4). These nerves also interact with the dorsal nerve of the penis at its base (15-17). Anatomical dissections suggest continuity between the bulbospongiosus and the superficial portion of the external anal sphincter, both in structure and innervation, indicating potential functional overlap (16).

Schematic drawing shows the innervation of BSM.

1) Anterior muscular branch of pudendal nerve; 2) Posterior muscular branch of pudendal nerve, the muscular branches supply the perineal muscles, including the BSM; 3) Isquiocavernosum muscle and 4) Superficial transverse perineal muscle.

### Anatomical Variations of Bulbosponjosum Muscle

Although the anatomy of the BSM is classically described, anatomical and clinical literature has documented several variations that may have functional and surgical implications. These variations can involve the origin, insertion, morphology, and even the presence of accessory muscle fibers or fusion with adjacent muscles (11, 19).

One of the most notable variations concerns the extension of BSM fibers. In some individuals, the anterior fibers may extend beyond what is typically described, reaching the pubic bone and terminating in a tendinous expansion that envelops the dorsal penile vessels. This variation may influence erection dynamics and venous compression, thereby contributing to penile rigidity (4).

Another important variation involves the continuity of the BSM with other perineal muscle structures. Anatomical dissections and advanced imaging studies, such as diffusion tensor magnetic resonance imaging, have suggested a continuity between the bulbospongiosus and the superficial portion of the external anal sphincter. This continuity may be both structural and neural, indicating a potential functional overlap and a more integrated perineal muscular network than previously thought. The presence of smooth muscle bundles interposed between the external anal sphincter and the bulbospongiosus, continuous with the longitudinal smooth muscle of the rectum, further supports the idea of a coordinated role in pelvic floor dynamics (2, 4, 13).

Variations in the insertion of intermediate BSM fibers have also been observed, with some fibers interweaving with contralateral fibers over the dorsal surface of the corpora cavernosa, forming a robust aponeurosis. This configuration may directly impact the muscle's ability to compress the erectile tissue of the bulb and assist in erection.

Moreover, the morphology of the BSM itself has been a subject of debate in scientific literature, with some descriptions indicating greater variability in the shape and size of the muscle among individuals. This variability may be influenced by genetic, ethnic, and even physical activity factors. Understanding these variations is crucial for urologic surgeons and other healthcare professionals working in the perineal region, as they may impact surgical planning and functional outcomes of procedures involving the pelvic floor.

In summary, anatomical variations of the bulbospongiosus muscle represent an ongoing field of study that offers important insights into the complexity of male perineal anatomy and its implications for urological function and surgical practice.

### Bulbosponjosum Muscle Histology

Histologically, it is predominantly composed of skeletal striated muscle fibers, interspersed with collagen and elastic fibers, as well as a well-developed vascular network.

Morphometric analyses have shown that, in healthy individuals, both the diameter of the muscle fibers and the relative proportion of collagen and elastic fibers tend to remain consistent. However, variations in vascular density may occur under certain pathological conditions. The perineal fasciae surrounding the muscle also contain collagen and elastic fibers, and there is evidence that the muscle can be subdivided into ventral and dorsal portions, with variable amounts of interposed connective tissue between them (9, 20).

Experimental studies in animal models confirm the predominance of striated muscle fibers in the BSM and suggest that fiber-type composition may undergo changes in response to specific physiological conditions, such as multiparity. However, these findings are mostly limited to non-human species, which restricts their direct applicability to human anatomy (9).

### Clinical and Surgical Implications of Bulbospongiosus Muscle

The BSM plays a crucial role in various male urological functions, and a thorough understanding of its anatomy and function is of paramount importance in clinical and surgical practice. The clinical and surgical implications of the BSM are extensive, ranging from sexual and urinary dysfunctions to complex reconstructive procedures.

### BSM and Ejaculation Process

The BSM is a striated muscle wrapped around the bulb of the urethra. Clonic contractions of the bulbocavernosus and other perineal muscles expel semen from the urethra. The BSM is innervated by the perineal nerve, a division of the pudendal nerve. This nerve containing afferent and efferent axons originates in a bundle coursing posterior to anterior towards the lateral aspect of the BSM muscle and sends branches across its surface (21). The perineal efferent innervation to the muscle is supplied from motoneurons in the pudendal nucleus located in the sacral segments of the conus medullaris. Its function is to propel seminal fluid from the urethra during ejaculation. During the initial phase of ejaculation, known as emission, secretions from the periurethral glands, seminal vesicles and prostate, along with sperm from the vas, are deposited into the posterior urethra and drain into the anterior urethra (21). Stimulation of the dorsal nerve of the penis leads to BSM contraction that provides an indication of anatomic sacral nerve pathway integrity (22, 23), or through electrostimulation (24-26).

The stimulus of seminal fluid in the urethra, with afferent impulses mediated through the DNP, provides positive feedback to the pudendal nuclei in the sacral spinal cord during ejaculation. This sensory input acts to initiate and maintain BCM contractions until the urethra is emptied of semen (21). These contractions cannot be elicited by passage of urine through the urethra. Urine in the anterior urethra is not analogous to the passage of semen following sexual arousal and thus would not result in BSM contraction. With sexual arousal, the presence of circulating neurohormonal agents modify synaptic transmission, resulting in coordinated contractions of the BSM. The sensation of orgasm may be mediated through the pudendal fibers (27).

**Figure 5:**
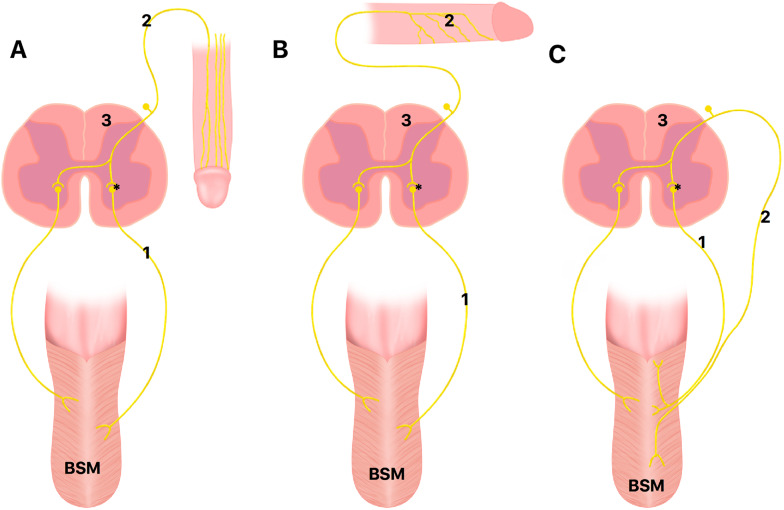
Somatic reflexes of the of Bulbospojosum muscles (BSM).

The BSM reflex were evoked from all segments of the anterior urethra (bulbus, penile, glandular), as well as from the penile skin, and the responses varied depending on the point of stimulation (21). The response from the DNP at the penile base was rapid, consistent and had a characteristic morphology (Figure-5). At the penile base, the DNP is a large, single trunk on either side of the mid-line with minimal branching, so stimulation applied at this point would be most likely to result in a clear response. The response at the glans was of longer latency, but still displayed the same wave pattern. The increase in latency can be attributed to the increase in nerve length from the penile base to the glans, and that there are smaller numbers of DNP fibers being stimulated at the glans. In addition, the DNP within the glans is arborized into smaller branches and may have a higher depolarization threshold. The three distinct peripheral reflexes controlling ejaculation suggests the possibility of different central nervous system pathways, with conus medullaris control of ejaculation more complex than originally believed. All three afferent pathways synapse on pudendal motoneurons in the conus medullaris and provide for peripheral reflex control of BSM contractions.

(A) Schematic drawing showing the fibers of BSM via the perineal nerve (1) and the synapse (*) with dorsal penile nerve branches (2) by motoneurons in the pudendal nucleus in conus medullaris (3); (B)) Schematic drawing showing the ventrolateral branches of dorsal penile nerve (2) innervating the anterior urethra and the synapse (*) in the pudendal nucleus in conus medullaris (3), 1 – perineal nerve and (C) Schematic drawing showing the afferent (1) and efferent (2) axons in the perineal nerve innervating the BSM, * - synapse in the pudendal nucleus in conus medullaris (3).

### BSM and Urethroplasty

Bulbar urethroplasty can evolve with ejaculatory dysfunction and post-micturition dribbling, which has led to the preservation of BSM during the surgical procedure. The preservation of BSM and its innervation during bulbar urethroplasty could improve the ejaculatory function in the postoperative period.

Several theories have been proposed to explain the exact cause of ejaculatory dysfunction and post voiding dribbling after bulbar urethroplasty: injury of the perineal nerves; injury of the BSM; alteration of the structure of the bulbar urethra and the bulbourethral reflex; sacculation formation after bulbar urethroplasty (buccal mucosa graft support leading a sacculation) and injury of the vessels that supply the bulbar urethra and BSM (4, 28-31).

Previous studies have shown that the preservation of the BSM and perineal nerves during bulbar urethroplasty are safe, viable and minimally invasive technique with minimal effect on the patient's ejaculatory function (29, 30, 32, 33).

Previous studies have evaluated the histology of bulbar urethral stricture and concluded that it is characterized by changes in extracellular matrix characteristics (34-37).

**Figure 6:**
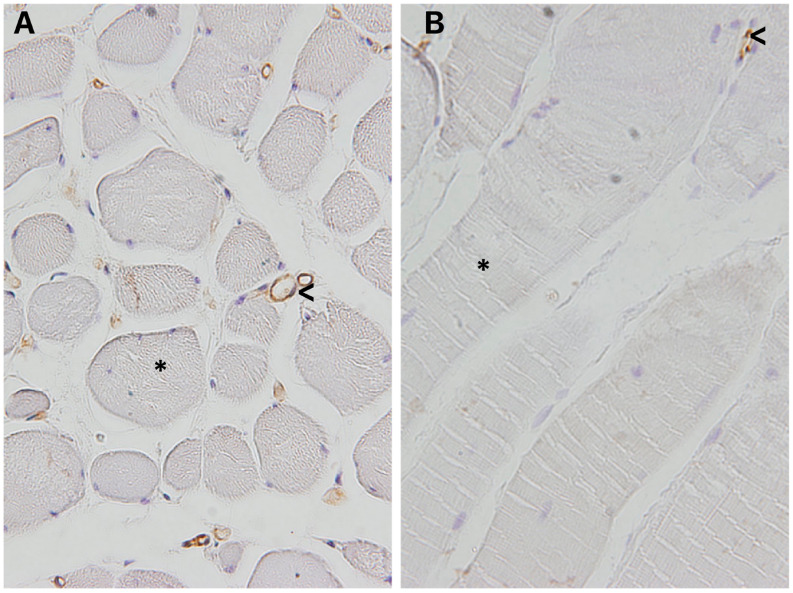
Histology of Bulbospojosum muscles (BSM) in patients submitted to Urethroplasty.

Histological analysis of the BSM in patients with bulbar urethral stricture showed a significant decrease of the vessels, without changes in elastic fibers, collagen, nerves, and muscle fiber diameter (Figure-6) (9). These findings show that the bulbar urethral stricture causes minimal alterations in the structure of the BSM during the surgical procedure. Histological analyzes showed a significant decrease in only of vessels of the BSM in bulbar urethral strictures, showing that this condition causes minimal alterations in its structure (9).

A) BSM sample of patient without urethral stricture. We can observe the vessels distribution (arrowhead). Immuno-histochemistry with anti-CD31 X400 and B) BSM sample of patient submitted to bulbar urethral stricture surgery. We can observe a significant decrease in the vessels distribution (arrowhead). Immuno-histochemistry with anti-CD31 X400.

### BSM and Androgenic Activity

**Figure 7:**
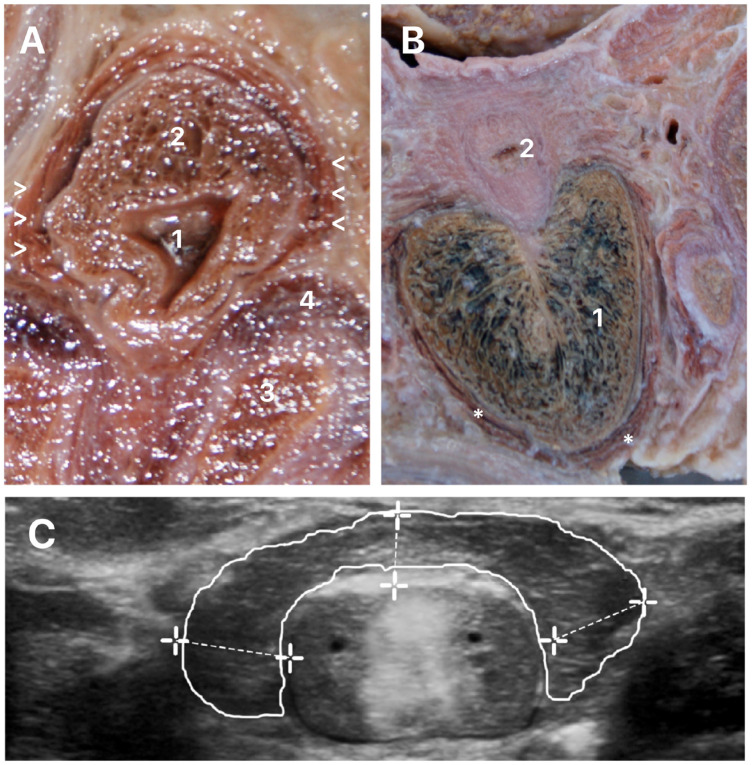
Measurement of Bulbospojosum muscles (BSM).

BSM is highly sensitive to androgen and could be used in common androgenic activity evaluation. The measurement of BSM is used for estimation of androgen tissue activity. Previous papers shows that the measurement of BSM is performed with the patient in the supine position, with slight frog-legged position of the lower extremities (38). The measurement is performed with a Transperineal ultrasound. The ultrasound measures the BSM length and thickness. In Figure-7 we can observe a schematic drawing of the BSM measurements. The BSM area was calculated with the patient in the same position with the stretched penile length (PL) and erect PL measured from the base of the dorsal surface of the penis to the tip of the glans (38). Ultrasound assessment of the BSM is an effective method to evaluate end-organ activity of androgens.

A) Transverse section of fresh frozen male cadaver showing the relationship between the urethra (1), corpus sponjosum (2) and BSM (arrowhead), 3- corpus cavernosum, 4- Tunica albuginea; B) Frontal section of male fresh frozen cadaver showing the relationship between the sponjosum tissue (1) and BSM (arrowhead), 2- Urethra and C) Schematic drawing based in paper of Dabaja (38) showing the measurement of BSM length and thickness with a Transperineal ultrasound.

## CONCLUSIONS

The BSM is a paired skeletal striated muscle that originates from the perineal body enveloping the bulbar portion of the urethra. Stimulation of the dorsal nerve of the penis leads to BSM contraction that provides an indication of anatomic sacral nerve pathway integrity. BSM has some important clinical applications. The bulbar urethral stricture causes minimal alterations in the structure of the BSM during the surgical procedure and the BSM is highly sensitive to androgen and could be used in common androgenic activity evaluation.

## Data Availability

Data will be available upon request.
